# Estimating the number of children in households with substance use disorders in Germany

**DOI:** 10.1186/s13034-021-00415-0

**Published:** 2021-11-06

**Authors:** Ludwig Kraus, Alfred Uhl, Josefine Atzendorf, Nicki-Nils Seitz

**Affiliations:** 1 IFT Institut für Therapieforschung, Leopoldstraße 175, 80804 München, Germany; 2 Department of Public Health Sciences, Centre for Social Research On Alcohol and Drugs, Stockholm University, Stockholm, Sweden; 3 Institute of Psychology, ELTE Eötvös-Loránd-University, Budapest, Hungary; 4 Austrian Public Health Institute, Vienna, Austria; 5 Sigmund Freud Private University, Vienna, Austria; 6 Munich Center for the Economics of Aging (MEA), Max-Planck-Institute for Social Law and Social Policy, Munich, Germany

**Keywords:** Children, Adolescents, Parental substance misuse, Estimation, Germany

## Abstract

**Background:**

Parental substance misuse is reported to endanger the health and psychological development of children and adolescents. The aim of the present study was to address conceptual and methodological problems in estimating the number of children affected by parental substance misuse (CaPSM) and offer a novel approach based on survey data.

**Methods:**

Data came from the 2018 German Epidemiological Survey of Substance Abuse (ESA) among 18- to 64-year-olds (n = 9267) and from population statistics. DSM-IV diagnostic criteria were used to assess substance use disorder (SUD) related to tobacco, alcohol, cannabis, cocaine or amphetamine. Based on the number of household members, the number of children below age 18 years and the information on SUD status of the respondent living in this household, the number of children currently living in households with at least one member with SUD was estimated.

**Results:**

In 2018, there were 13,597,428 children younger than 18 years living in Germany. Of these, 6.9–12.3% (935,522–1,673,103) were estimated to currently live in households where at least one adult had a tobacco use disorder, 5.1–9.2% (688,111–1,257,345) in households where at least one adult had an alcohol use disorder and 0.6–1.2% (87,817–158,401) in households where at least one adult had a disorder related to the use of illicit drugs. The total number of children currently living with SUD adults in their household was estimated at 11.2–20.2% (1,521,495–2,751,796).

**Conclusions:**

Available estimates are difficult to interpret and to compare due to a lack of clear case definitions and methodological approaches with various biases and limitations. Future estimates need to provide precise case definitions and standard approaches.

## Background

Parental substance misuse (PSM) is reported to endanger the healthy development of children and adolescents. These children and adolescents are at increased risk of negative outcomes such as emotional, social and behavioural adjustment problems as well as deficits in cognitive and academic functioning [1–3]. They also face a higher risk of early substance use involvement as well as mental health problems [4, 5]. For instance, anxiety disorder [6], depression [7, 8], attention-deficit/hyperactivity disorder [9, 10] and also disturbed social behaviour [11, 12] were more common in the children and adolescents of parents with substance use disorder (SUD) compared with the offspring of parents without SUD.

PSM is often associated with problematic parenting, mood swings, disinhibited behaviour and unmet parental responsibilities [13, 14]. Older children of substance-misusing parents must sometimes step in to fulfill the role of their parents such as feeding a baby or taking care of younger siblings while parents experience withdrawal symptoms or recover from a hangover [15]. Parental and family problems often lead to an atmosphere in the home of fear, chaos, uncertainty, secrecy and stigma of living with these problems [16]. Furthermore, unstable parent–child relationships resulting from separation from or death of a parent, conflicts, aggression and violence in the family aggravate these adverse living conditions [17, 18].

### Theoretical considerations

Estimating the number of children affected by parental substance misuse (CaPSM) constitutes a major challenge on account of the definition of exposure. A lifetime approach assesses how many children were exposed to substance-misusing (SM) parents or other carers during childhood and adolescence, resulting in a prevalence until maturity. This can be achieved using a retrospective approach by asking young adults older than, for instance, 18 years about their experience with PSM during childhood and adolescence. The number of people who were affected by PSM in the course of their childhood and adolescence as well as the number of people not affected can be directly assessed and the percentage of children affected by PSM can be calculated (see [19, 20]). However, such approaches need to account for the time-varying nature of exposure, e.g. parents’ or carers’ substance use may be episodic, or they may episodically be present and absent from their children's lives as a result of parental desertion, child removal or otherwise. Conversely, a current approach quantifies the number of CaPSM who are exposed to PSM by parents or other adults they are presently cohabiting with (see, [21, 22]).

Although the majority of children live with their biological parents, estimates including only biological parents neglect potentially adverse conditions of children living with social parents with substance use problems, or where significant others they are living with are misusing substances (see [23]). Social parents may be step, adoptive or foster parents, grandparents or other relatives. Other cohabiting adults who are not legal guardians may be new partners of a parent, grandparents, older siblings, other relatives or other people within a flat- or house-sharing community (see [22]). However, many biological parents, social parents or significant others with substance use problems never lived in the same household as their minor offspring or do not live with them anymore, but may nevertheless have affected the child’s wellbeing.

Of similar importance are the pattern and severity of substance use problems considered. What is labelled ‘excessive’ or ‘problematic’ substance use is much more prevalent than ‘pathological’ substance use, in the sense of SUD (see [22]). Thus, the estimated number of children at risk of experiencing harm is highly dependent on the type of exposure considered as potentially causing harm. Yet another crucial aspect is the particular substance the parent or carer is misusing. The impact of parental dependent tobacco smoking on children is obviously very different from the impact of misusing alcohol or using strongly intoxicating and mind-altering substances [13, 14, 24].

Thus, the existing estimates of the number of CaPSM broadly differ in terms of the chosen definitions of exposure and, consequently, estimates accounting for exposure during childhood and adolescence will be higher than measures of current exposure, and restricting exposure to biological parents will result in lower estimates than a definition including socially transmitted risks from close relatives or significant others living in the same household. Finally, defining exposure as excessive substance use or SUD of parents or cohabiting adults will clearly affect the estimate.

### Methodological considerations

The majority of the rather few estimates of the number of CaPSM take the perspective of current rather than lifetime exposure and are based on household surveys. By selecting households and collecting all necessary information on adult household members and the number of children living with them, the number of children currently affected by SM adults as well as the number of children not affected can be estimated [22, 23, 25–27]. Of these surveys, the majority are based on an individual household member approach (IHMA) where only one adult in a selected household is interviewed [22, 23, 25, 27]. Based on a SM assessment of the selected respondent, the number of children living in the household and information on the respondent–child relationship, the number of CaPSM has been estimated. As the SM status of other adult members living in the same household as the respondent is usually not assessed in household surveys, the number of CaPSM using the IHMA approach assumes that there is no more than one adult with SM status living in the household.

Information on the number of children affected by SUD of adults is needed in order to address specific needs for health care initiatives and professional as well as non-professional support. A significant caveat of surveys based on the prevailing IHMA approach is the implicit assumption that only one adult per household may be affected with SUD, resulting in an overestimate due to multiple counting in cases where there is more than one adult affected by SUD. In the present paper, we offer a novel approach to overcome this limitation by assuming two extremes when identifying a SUD-positive adult in a household: only one adult is or all adults are affected by SUD. This results in a lower and upper threshold of the number of CaPSMs. As case definition of CaPSM, we focus on current SUD problems of cohabitating adults including biological and social parents as well as other adults living in the same household, such as older siblings. Parents not currently living in the household are not considered. Children or adolescents under age 18 years living in the same household are defined as the target population. Although any substance use may affect a person’s control over emotions, judgements or behaviour negatively, we restrained our estimates to the more severe conditions of SUD as defined by the DSM-5 criteria [28]. The psychoactive substances considered in relation to SUD were tobacco, alcohol and illicit substances such as cannabis, cocaine or amphetamine.

## Methods

### Data

Estimates were based on data from the German Epidemiological Survey of Substance Abuse (ESA) conducted in 2018 [29] and on data on population statistics [30]. The sample includes German-speaking individuals aged between 18 and 64 years living in private households. A multistage sample selection was performed: first, 254 municipalities (sample points) were randomly selected followed by a random selection of the target population from population registers. Data were collected by standardized self-administered questionnaires (paper–pencil), telephone interviews or online questionnaires. The adjusted sample included 9267 individuals (response rate: 41.6%). Details on the methods and design of the ESA 2018 have been published elsewhere [27].

In the section on demographics in ESA 2018, participants were asked about both the number of children under the age of 18 years and the number of adults including the respondent who currently lived in their household. Moreover, information about the family status of the respondents (married/partnership, divorced, widowed), whether children were present and the relationship with each child (biological, step, adoptive or foster parent, brother or sister, nephew or niece, other) was collected. SUD related to tobacco, alcohol, cannabis, cocaine or amphetamine was assessed using the criteria of the DSM-5 at the respondent level, but no information about the SUD status of other adults in the household was collected (IHMA approach). Diagnoses were based on the Munich Composite International Diagnostic Interview (M-CIDI; 31, 32).

### Analysis

The analysis was based on the study participants’ information on the number of people living in their households, the number of children below age 18 years living in these households and information on the participants’ SUD status. Owing to the low number of individuals fulfilling criteria for disorders related to cocaine (n = 16) or amphetamine (n = 28), these disorders were combined with cannabis-related disorder and labelled ‘illicit substance use disorders’. As the sample represents individuals in the adult population, it is assumed that the results are proportional to a full census covering the target population. In a full census, every household with more than one adult would result in the same children being counted multiply as they are related to more than one adult. Children indicated by an SUD-affected adult would be considered to be CaPSMs and, if they are indicated by another adult from this household without a SUD problem, they would be counted erroneously as non-CaPSMs. For instance, considering six children in a household with three adults and adding up the number of all children would incorrectly result in 18 children, even though there are actually only six. To avoid multiple counting, the share of children per adult needs to be considered. By dividing the number of children related to each respondent by three (share of children per adult), the total sum over all adults represents the correct number of children in each household 6/3 + 6/3 + 6/3 = 6.

When counting the number of SUD-affected children based on the number of children reported by the adults in each household, the fundamental problem arises that children living with a surveyed non-SUD-affected parent are counted as non-CaPSM even though they may be living in a household with another SUD-affected adult and therefore should be classified as CaPSM. As the SUD status of other adults per household was not assessed in the ESA, there is no precise way to compensate for this shortcoming. However, it is possible to calculate a lower and an upper point estimate for the number of children affected by SUD in households by applying the two most extreme diametrical assumptions:

- Assumption 1: All surveyed respondents, regardless of being SUD-positive or SUD-negative, cohabit exclusively with adults with the same SUD status. If this is the case, all children in the population are correctly classified as SUD affected or not.
- Assumption 2: No more than one adult in each household is affected by SUD. In this case, all children mentioned by SUD-negative adults in households with SUD-positive adults are erroneously classified as ‘non-SUD affected’.

For each individual in the sample, three variables were used: the number of children in the household (${n}_{c}$), the number of adults in the household (${n}_{a}$) and the SUD status $i$ of the surveyed adult ($i \in \left\{SUD, nonSUD\right\}$). Based on Assumption 1 and adding the share of children per adult for all households, the number of SUD-affected children (${N}_{SUD.1}$) and the number of non-SUD-affected children ($N_{nonSUD.1}$) was calculated:

$$N_{SUD.1} = \mathop \sum \limits_{i = SUD} \overline{n}_{c} \cdot n_{a}$$

$$N_{nonSUD.1} = \mathop \sum \limits_{i = nonSUD} \overline{n}_{c} \cdot n_{a}$$

Under Assumption 1, adding the shares of children for all SUD adults represents the correct number of children affected by SUD in the households, as Assumption 1 rules out the possibility that SUD-negative adults live in the same households with SUD-positive adults. For example, if there are six children living in a household with three SUD-positive adults, the share of children for each adult is two and all children are CaPSMs. Summing the share of children for the three SUD-positive adults adds up correctly to six affected children (3*2 = 6).

Under Assumption 2, we are confronted with the situation in which the shares of children linked to SUD-negative adults who are actually affected by other SUD-positive adults living in the same households are misclassified. Assuming that there is no more than one SUD-positive adult in every household, all other adult household members must be SUD negative. The total number of misclassified shares of children (adjustment term) needs to be subtracted from the number of initially (in line with Assumption 1) classified unaffected children $N_{nonSUD.1}$ and added to the number of children initially classified as affected $N_{SUD.1}$. For example, if there are six children in a household with two non-SUD adults and one SUD adult, all six children are affected by SUD, but only the SUD adult’s children share is initially considered as affected (1*2 = 2), even though six children are actually affected. The difference of four children must be compensated for to arrive at correct estimates. Using the adjustment term

$$N_{adj} = \mathop \sum \limits_{i = SUD} \overline{n}_{c} \cdot (n_{a} - 1)$$

the number of SUD-affected children ($N_{SUD.2}$) and the number of non-SUD-affected children ($N_{nonSUD.2}$) in line with Assumption 2 were calculated as follows:

$$N_{SUD.2} = N_{SUD.1} + { }N_{adj}$$

$$N_{nonSUD.2} = N_{nonSUD.1} - { }N_{adj}$$

The percentage of affected children $\frac{{N_{SUD} }}{{N_{SUD} + N_{nonSUD} }}$ reflecting the proportion of SUD-affected children among all children was calculated under both assumptions. Under Assumption 1, $\frac{{N_{SUD.1} }}{{N_{SUD.1} + N_{nonSUD.1} }}$ yields the lower point estimate and, under Assumption 2, $\frac{{N_{SUD.2} }}{{N_{SUD.2} + N_{nonSUD.2} }}$ yields the upper point estimate.

Lower and upper point estimates of $N_{SUD.1}$ and $N_{nonSUD.1}$ as well as $N_{SUD.2}$ and $N_{nonSUD.2}$ were calculated separately for the conditions of tobacco, alcohol and illicit substance use disorders. Multiplying the obtained point estimates by the number of children younger than 18 years in the population (N = 13,597,428 as of 31 December 2018; [30]), the range in the number of children living in households with at least one member having a positive SUD diagnosis was projected to the total population. All analyses based on survey data were performed using Stata 15.1 (Stata Corp LP; College Station, TX, USA).

## Results

The 12-month prevalence of SUDs is shown in Table 1. The prevalence of SUDs in adults ranged between 1.6% for illicit drug use disorder, 8.5% for alcohol use disorder and 15.5% for tobacco use disorder.

**Table 1:** 12-month prevalence of substance use disorders according to DSM-5 among 18- to 64-year-olds

Substance	n	%	[95% CI]^a^
Tobacco	1140	15.5	[14.4; 16.7]
Alcohol	973	8.5	[7.8; 9.2]
Illicit drugs^b^	176	1.6	[1.3; 2.0]

^a^95% confidence interval

^b^cannabis, cocaine or amphetamine

The family status of SUD and non-SUD families differed statistically significantly. More than two thirds (72.5%) of non-SUD respondents were married or had a partner, whereas the proportion was significantly lower in respondents with SUD status (49.4%) and lowest among respondents with illicit substance use disorder (20.0%; Table 2). On average, 1.7 children were living in non-SUD households and 1.6 in SUD households. In non-SUD households, 85.0% of children lived with biological parents, whereas in SUD households, 68.1% did; this difference was statistically significant (p ≤ 0.01). The proportion of single parent/carer families was lower in non-SUD households (6.1%) than in SUD households (7.5%), but the difference did not reach statistical significance.

**Table 2:** Sample characteristics in households with children by adult SUD status: number of adults in household, age of respondent, family status of respondent; number of children in household and relationship with adult

	Non-SUD		SUD		Tobacco		Alcohol		Illicit drugs^c^	
	n = 2448		n = 507		n = 318		n = 238		n = 31	
Adults										
N in household (Mean, SE^a^)	2.2	0.02	2.2	0.04	2.2	0.05	2.3*	0.05	2.4	0.16
Age (Mean, SE)	41.1	0.17	38.2**	0.47	39.0*	0.57	35.9**	0.84	30.4**	3.44
Family status (n, %^b^)			****		****		****		***	
Not married	590	24.2	230	49.4	121	38.3	141	59.7	23	76.7
Married/partnership	1,770	72.5	249	49.4	174	38.3	90	38.1	6	20.0
Divorced/widowed	83	3.4	25	5.0	21	6.6	5	2.1	1	3.3
Missing	5		3		2		2		1	

	n = 4024		n = 789		n = 509		n = 348		n = 44	
Children										
Age (Mean, SE)	9.7	0.11	9.8	0.26	9.7	0.32	9.9	0.49	7.5*	1.01
N in household (Mean, SE)	1.7	0.02	1.6	0.05	1.6	0.06	1.5*	0.07	1.7	0.19
Living with one adult (n, %)	121	6.1	38	7.5	32	10.1	9	5.0	2	6.5
Relationship with adult (n, %)			****				****			
Biological parent	3,384	85.0	529	68.1	381	76.8	183	52.6	18	41.9
Step/adoptive/foster parent	75	1.9	21	2.7	16	3.2	8	2.3	–	–
Brother/sister/ nephew/niece	485	12.2	212	27.3	92	18.5	148	42.5	25	58.1
Other	35	0.9	15	1.9	7	1.4	9	2.6	–	–
Missing	45		12		13				1	

^a^Standard error

^b^% of valid cases

^c^Cannabis, cocaine or amphetamine

*adjusted Wald test with non-SUD, p ≤ 0.05; **adjusted Wald test with non-SUD, p ≤ 0.01; ***Chi^2^ test with non-SUD, p ≤ 0.05; ****Chi^2^ test with non-SUD, p ≤ 0.01

The estimates of the number of children in households with adults affected by SUD and the prevalence of affected children among all children are presented in Table 3. The results based on single substance disorders of adults suggest that the highest proportion of affected children live in households with at least one adult with a tobacco use disorder (lower and upper estimate: 6.9–12.3%). The proportion of children living in households with at least one adult affected by alcohol use disorder range between 5.1 and 9.2%, and between 0.6 and 1.2% of children live in households with at least one adult with illicit drug use disorders. Considering double and multiple SUDs but excluding tobacco use disorder, we estimated between 5.3 and 9.8% or 726,624 and 1,327,223 affected children. Including tobacco use disorder, a total of 11.2–20.2% or 1,521,459–2,751,796 children were estimated to live in households with at least one SUD-affected adult.

**Table 3:** Estimated number of children in households with at least one adult affected by type of substance use disorder and prevalence among all children below age 18 years

Substance	Estimated number of affected children (N)		Prevalence among all children (%)^a^	
	Lower limit	Upper limit	Lower limit	Upper limit
Tobacco	935,522	1,673,103	6.9	12.3
Alcohol	688,111	1,257,345	5.1	9.2
Illicit drugs^b^	87,817	158,401	0.6	1.2
Total				
Excluding tobacco	726,624	1,327,223	5.3	9.8
Including tobacco	1,521,495	2,751,796	11.2	20.2

^a^13,597,428 children aged < 1 to 17 years; Population Statistics 2018 (Statistisches Bundesamt (Destatis) 2019)

^b^Cannabis, cocaine or amphetamine

## Discussion

Using a representative survey of adults and information on their SUD status as well as the number of adults and children currently living in the same household with the respondent, the number of SUD-affected children cannot be precisely estimated. Not knowing the SUD status of non-surveyed adults in the household, summing the number of children reported by each respondent would overestimate the total number of children as a result of multiple counting. This problem can be solved by calculating the share of children per adult and by assuming that, whenever a surveyed adult is SUD affected: (1) all other adults in the household are SUD positive as well or (2) all other adults are SUD negative. The first assumption results in a conservative lower point estimate of SUD-affected children, whereas the second assumption results in an upper point estimate by both aggregating the share of children per adult for every household (dividing the number of children by the number of adults) and adjusting for children misclassified as non-CaPSM because the person interviewed was SUD negative even though another adult in the household was SUD positive.

It is important to note that the cross-sectional perspective of childrens’ current exposure to SUD taken in the present approach misses all occasions related to SUD cohabiting adults before the assessment and all occasions that will occur later until the children are of age. In comparison with this, if adult respondents were asked whether, during their childhood and adolescence, they were affected by the substance use of a parent or another adult they were closely related to, a retrospective approach including all past experiences would be taken resulting in a higher number of affected children.

### Comparisons with national and international estimates

In 2018, the number of children below the age of 18 years in households with SUD in Germany was estimated at 112–202 per 1000 children. Excluding exposure to tobacco use disorder, the result was 53–98 per 1000 children. Compared with an earlier estimate of 2.65 million children who were ever living with parents with alcohol use disorder by Klein [33], our estimate (688,111–1,27,345) is considerably lower. Naturally, asking 14- to 24-year-olds from a community sample about lifelong exposure to parental alcohol use problems [25] will result in a higher estimate. The second German estimate of 6.6 million affected children is based on the approach described above but implicitly only on Assumption 1 [34]. Moreover, different from our definition, the authors of this study used the AUDIT-C, i.e. a score of five or more points for men and four or more points for women, to define alcohol use problems. Using less severe criteria of risky drinking in terms of frequency and quantity of drinking and frequency of heavy drinking occasions naturally increases the number of exposed children.

Comparisons with international estimates are similarly limited because of different definitions of exposure and substance use problems among parents, carers or adults living in the same household. The proportion of children under 20 years with one or both parents misusing alcohol was estimated at 10.7% in Denmark, 5.7% in Finland, 15.4% in Germany and between 17 and 23% in Poland [35]. Alcohol misuse by parents was labelled ‘alcohol problems’ in Denmark, ‘excessive alcohol use’ in Finland, ‘alcoholism’ in Germany and ‘alcohol addiction’ or ‘alcohol abuse’ in Poland. Similarly, estimates for the proportion of children under 20 years with one or both parents using drugs was 0.2% in both Denmark and Germany and 1.5–2.4% in the UK. The definition ranged from drug use in Denmark to drug dependency in Germany and serious drug problems in the UK. Using a similar definition to the one in our study and conducting secondary analyses of data from national household surveys, a study in the UK estimated the proportion of children below the age of 12 years currently living in a household with an alcohol-dependent adult at 5.9% and children currently living in a household with a drug-dependent adult at 2.8% [22]. Grant [26] used data from the 1992 National Longitudinal Alcohol Epidemiological Survey and calculated the rate of children aged 17 years or younger living in households with one or more adults who were abusing or dependent on alcohol (last 12 months) and who, at some time in their lives, had abused or were dependent on alcohol, resulting in a lower (15%) and upper estimate (43%). Excluding non-parental relationships with the child in the household, Bassani and colleagues [23] estimated that 11.4% and 8.3% of children aged under 12 years in Canada were exposed to SUDs and alcohol use disorders (i.e. excluding illicit substances) of their biological parents respectively.

### Potential risks of harm

Although our findings suggest that between 11.2 and 20.2% of all children in Germany are living in households with at least one adult with any SUD, these children may be subject to different risks depending on the type and severity of the disorder. For instance, children living in households where adults smoke have a higher risk of somatic diseases such as asthma and other respiratory conditions [36, 37], whereas children exposed to intoxicated cohabitants are more likely to face psychologically stressful situations leading to lower school performance or behavioural problems [38–40]. This can be neglect, aggression or having to take over parental roles. Children of parents with SUD generally have a higher risk of drug involvement as well as mental health problems or disturbed social behaviour compared with the offspring of parents without SUD [4, 41]. Research also indicates that these children are at higher risk of developing SUDs themselves, as well as non-substance-related psychopathologies [42].

Respondents with SUD in our study were more often living in single parent/carer households compared with non-SUD respondents, indicating the need for supportive strategies for these parents and children. There is evidence that young people raised by single parents are more likely to perform poorly in school and partake in deviant behaviours such as smoking, substance use and crime [43].

It is also important to point out that estimates of the number of children affected by parental SUD indicate a potential risk of adversity. Quantitative and qualitative aspects, such as the frequency and intensity of the adults’ problematic SUD-related behaviours, are not considered in any study. For instance, some children may be exposed to SUD adults in their household who often behave violently when intoxicated, or who are depressed and stress their children with suicidal ideas. Others may live with adults who primarily damage their own physical health through excessive alcohol or drug consumption and only slightly affect the children in their environment. Despite experiencing negative somatic, psychological or social consequences, some children do not show signs of negative psychological developments on account of factors of resilience supporting these children in developing stable and assertive personalities [44–47].

### Notes on prevention

There is ample evidence that parents and carers of children with SUD have strong and often irreversible negative somatic and psychological effects on the wellbeing of their children. To target these children, prevention programmes and policies have been developed [15, 48, 49]. For instance, secondary prevention targeting individual, familial and environmental influences by offering specific help to affected children and parents has been proven effective in reducing future problems in these children [50]. In addition to providing preventive support for parents with SUD and affected children, the harmful effects of SUD on others and particularly on children need to be recognized as a public health concern in the same way as are the harmful effects on the users or the costs to society [51].

### Strengths and limitations

To account for multiple counting of children in households where more than one adult lives, we based our calculations on the share of minors per adult in every household. Under Assumption 1, aggregating these shares per SUD adult and non-SUD adult provides an estimate of the number of SUD-affected and non-SUD-affected children in the sample. Under Assumption 2, we compensated for the percentage of shares of children associated with non-SUD adults in households where children are affected by another SUD adult. Finally, the percentage of SUD-affected children could be calculated without having to resort to total population figures under both assumptions.

However, the availability of data on SUDs in our study was limited to disorders related to the use of tobacco, alcohol, cannabis, cocaine or amphetamine. These data may account for the majority of substance-related disorders in the population, but SUD coverage is not comprehensive. Estimates of SUDs from survey data are usually subject to underreporting because of higher non-response in particular subgroups or socially desirable response behaviour. For instance, surveys usually miss subgroups with higher risks of SUDs such as people who are homeless, in prison, hospitalized or living in institutions at the time of the survey. Moreover, biases may be caused by systematic non-participation. The ESA is subject to a ‘middle-class bias’, i.e. individuals with low socioeconomic status are under-represented, challenging the representativeness of the data [52]. Finally, household surveys are cross-sectional in nature with respondent substance use and parental status measured at a single point in time; yet these are not stable factors but fluctuate over time.

## Conclusions

To conclude, estimates of the number of CaPSM can vary substantially due to several aspects. The observation may refer to the total lifetime of children and youths until reaching adulthood, the period until the assessment or the current situation. The methodological approaches may assess alcohol use patterns of adults cohabiting with minors or ask adult CaPSMs about their experiences with SUD-positive parents or other adults cohabiting with them during childhood and adolescence. Assessments may consider the SUD status of the interviewed adult or the SUD status of other household members as well. Exposure may be defined as excessive use, problematic use or substance use disorder. Finally, respective SUD adults may be biological or social parents, and/or significant others, who are living in the same household, and/or biological parents living elsewhere. In order to identify changes in exposure to risk in relation to the child’s age, future approaches would benefit from longitudinal or cohort study designs with multiple and regular assessment intervals. Most importantly, future estimates need to provide precise case definitions, and standard approaches need to be developed that include unequivocal definitions and descriptions of methodological approaches to avoid or compensate for biases. Only if this has been realized can CaPSM estimates be sensibly interpreted and compared with estimates stemming from other studies.

## Data Availability

All datasets from the Epidemiological Survey of Substance Abuse (since 1980) are available for scientific purposes at GESIS Leibniz Institute for Social Research and can be requested there. Permission to access the data must be obtained from the IFT Institut für Therapieforschung in Munich, Germany.

## Abbreviations

CaPSM: Children affected by parental substance misuse
ESA: Epidemiological Survey of Substance Abuse
IHMA: Individual household member approach
PSM: Parental substance misuse
SM: Substance misuse
SUD: Substance use disorder
